# ORP5 promotes tumor metastasis via stabilizing c-Met in renal cell carcinoma

**DOI:** 10.1038/s41420-022-01023-3

**Published:** 2022-04-21

**Authors:** Li Song, Lin Zhang, Yun Zhou, Xiaotong Shao, Yuting Xu, Dongsheng Pei, Qingling Wang

**Affiliations:** 1 Department of Pathology, Xuzhou Medical University, Xuzhou, Jiangsu 221004 China; 2 Department of Radiation Oncology, Xuzhou Central Hospital, Xuzhou, Jiangsu 221000 China

**Keywords:** Renal cell carcinoma, Metastasis

## Abstract

ORP5, a lipid transporter, has been reported to increase the metastasis of several cancers. However, the potential mechanisms of ORP5 in renal cell carcinoma (RCC) remain unclear. In this study, we demonstrated that ORP5 was commonly overexpressed in tumor cells and tissues of RCC, and associated with tumor progression. Overexpression of ORP5 could promote RCC cells migration and invasion. In addition, the results suggested that the expression of ORP5 was favorably associated with c-Met expression, and ORP5 promoted RCC cells metastasis by upregulating c-Met in vitro and in vivo. Mechanistically, ORP5 facilitated the ubiquitination and degradation of c-Cbl (the E3 ligase of c-Met), and thus inhibited c-Met lysosomal degradation, which resulted in the stabilization of c-Met. In general, these findings revealed the role of ORP5 in contributing to tumorigenesis via upregulating c-Met in RCC.

## Introduction

RCC is the 10th most frequently diagnosed carcinoma in women and the sixth in men worldwide [1]. Among these, clear cell renal cell cancer (ccRCC) is the commonest, accounting for the majority of cancer-related deaths [2]. Since the early loss of function is uncommon in patients suffering from renal cell carcinoma, about one-third of patients have metastatic disease at diagnosis [3]. In addition, those with localized tumors have up to 40% risk of recurrence following complete resection [4]. Based on the refractory nature of metastatic renal cell carcinoma, it is imperative to explore the early diagnosis signs of RCC and understand the regulatory mechanism underlying RCC progression.

ORP5 (Oxysterol-binding protein-related protein 5) is a tail-anchored endoplasmic reticulum (ER) membrane protein that functions as a transporter of lipids among intracellular membranes. Through a reverse transport process that occurs at the membrane junction, ORP5 transfers phosphatidylserine from the ER to the cytoplasmic membrane and phosphatidylinositol 4-phosphate to the ER [5, 6]. Current studies provide evidence that ORP5 is regularly upregulated in several cancers, such as lung cancer, pancreatic cancer, etc. [7, 8]. Moreover, ORP5 can promote the invasion as well as migration of human tumor cells [9, 10]. However, the roles of ORP5 in RCC are still unknown.

c-Met (Mesenchymal-epithelial transition factor) is a receptor tyrosine kinase expressed on the surface of several types of epithelial cells and HGF/SF (ligand hepatocyte growth factor/scatter factor) is its ligand [11]. HGF/c-Met plays key roles in cell migration and invasion by activating several important signaling pathways comprising phosphatidylinositol 3-OH kinase (PI3K)/Akt and Ras/Raf/ mitogen activated protein kinase (MAPK)/ extracellular signal-regulated kinase (ERK) [12, 13]. Currently, considerable evidence supports the idea that c-Met is abnormally expressed in various malignant tumors. Furthermore, abnormal activation of the c-Met is known to facilitate cancer cells cytoskeletal changes, favoring migration, invasion, and ultimate metastasis.

It is shown that HGF/c-Met signaling is engaged in modulating the classical Epithelial-mesenchymal transition (EMT) process [14, 15]. Our data indicated that ORP5 could regulate the EMT process of RCC cells. Meanwhile, the idea that ORP5 can drive mTORC1/AKT signaling activation in certain cancer cells, which is one of the main downstream pathways of c-Met has been confirmed [11, 16]. Then, we found that ORP5 could activate mTORC1/AKT signaling in RCC cells. In addition, c-Met was highly expressed in RCC, and this expression was associated with significantly worse pathological features and overall survival, suggesting that overexpression of c-Met was a potential poor prognostic marker for RCC patients[17–19]. Consequently, we postulated that ORP5 might participate in the regulation of c-Met signaling.

C-Cbl, a member of the Cbl family of ubiquitin ligases, has emerged as a pivotal negative modulator of c-Met [20]. C-Cbl is attracted to the activated c-Met receptor, thereby inducing multi-ubiquitylation of c-Met. To the end, the multi-ubiquitylated c-Met-Cbl complexes degrade by targeted lysosomes [21, 22]. Furthermore, the protein levels of c-Cbl can also be modulated by ubiquitination [23, 24]. In addition, tyrosine phosphorylation of c-Cbl is crucial for auto-ubiquitination [23, 25].

The potential mechanism of ORP5 in RCC was investigated in our study. Our results indicated that ORP5 was upregulated in cells and tissues of RCC and facilitated migration of RCC cells in vitro and *vivo*. We provided further proof that ORP5 promoted the metastasis of RCC cells by upregulating c-Met protein levels. Studies on the mechanisms suggested that ORP5 promoted proteasome degradation of c-Cbl and reduced the lysosomal degradation of c-Met. These results generally revealed a novel role in mediating RCC metastasis and that ORP5 might be a prospective target for RCC therapy.

## Results

### ORP5 is highly expressed in RCC cells and tumor tissues and associated with tumor progression

**Fig. 1:**
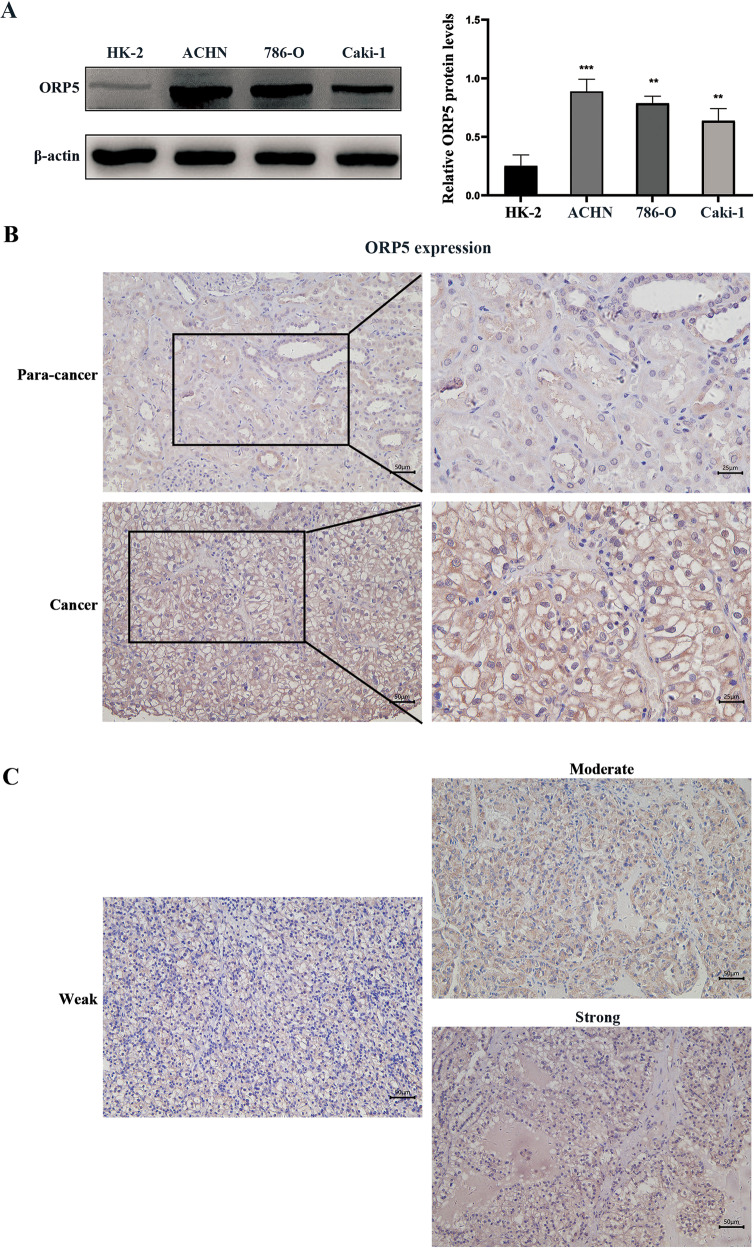
ORP5 is overexpressed in RCC cells and tumor tissues, and associated with tumor progression. **A** The expressions of ORP5 in several human RCC cell lines (ACHN, 786-O, and Caki-1) and human normal tubular epithelial cell line (HK-2) were measured by Western blot. Quantification of the ORP5 expression (right). **B** IHC assay of ORP5 protein expressions in para-cancer and cancer tissues in ccRCC patients. Scale bar, 50 µm (left) and 25 µm (right). **C** Representative images of IHC staining of ORP5 in RCC tissues. Scale bar, 50 µm. Error bars represent SD. **P < 0.01; ***P < 0.001.

Primarily, western blot analysis demonstrated that ORP5 was highly expressed in RCC cells comprising 786-O, ACHN, and Caki-1 compared with normal human renal tubular epithelial cell (HK-2) (Fig. 1A, *P* < 0.05). Then, we conducted IHC staining in tissue microarrays containing 75 tumor tissues of ccRCC to explore the correlations between ORP5 expression and ccRCC progression (Figs. 1B, C). The results indicated that ORP5 staining in cancer tissues of ccRCC was greater than that in para-cancer tissues.

Then, we evaluated the correlations between ORP5 expression and clinicopathological characteristics (based on TNM classification) in ccRCC. Statistical analysis suggested that high expression of ORP5 was associated with tumor size (*P* = 0.0372), pT status (*P* = 0.0422), and tumor stage (*P* = 0.0121) (Table 1). Altogether, the above results indicated that ORP5 was overexpressed in both RCC cells and tissues, and its increased expression was correlated with carcinogenesis and tumor progression.

**Table 1:** Clinical characteristics of 71 clear cell renal carcinoma patients and ORP5 expression.

Characteristics	Number (71)	ORP5 expression		*P* value
		High	Low	
All cases	71	50	21	
Gender				
Male	45	32	13	>0.9999
Female	26	18	8	
Age (years)				
<55	23	16	7	>0.9999
≥55	48	34	14	
Tumor size (cm)				
≤7	40	24	16	0.0372*
>7	31	26	5	
pT status				
pT1	28	16	12	0.0422*
pT2	23	21	3	
pT3	4	4	0	
pT4	15	9	6	
TNM stage				
I–II	49	30	19	0.0121*
III–IV	22	20	2	

Pearson’s Chi-square test and Fisher’s exact test were used.

*P < 0.05 was considered statistically significant.

### ORP5 promotes migratory and invasive capacities of RCC cells

**Fig. 2:**
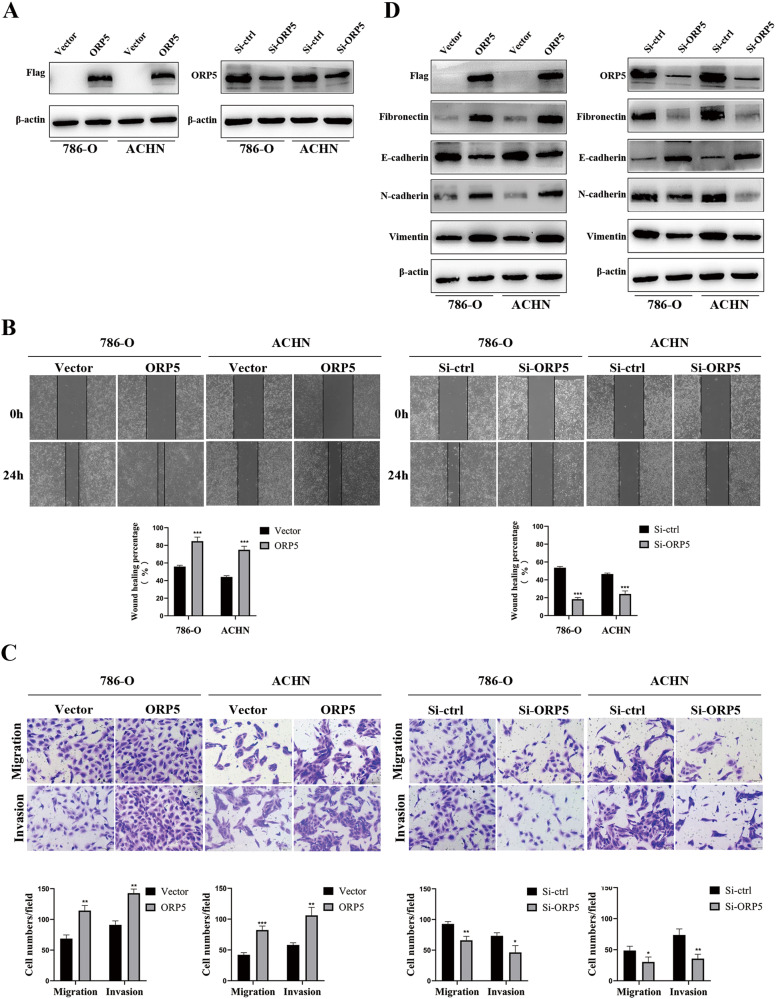
ORP5 promotes migratory and invasive capacities of RCC cells. **A** The protein levels of Flag and ORP5 in RCC cells with ORP5 knockdown or ORP5 overexpression were examined by western blot analysis. **B** The effect of ORP5 on cell motility was assessed by testing wound closure after upregulation or knockdown of ORP5 in RCC cells. Original magnifications, ×100 for (**B**). **C** Transwell assays were used to detect the changes in the migratory and invasive capacities of 786-O and ACHN cells after overexpression or downregulation of ORP5. Original magnifications, ×200 for (**C**). **D** The protein expressions of ORP5, N-cadherin, E-cadherin, Vimentin and Fibronectin in 786-O and ACHN cells after specific treatment were tested by Western blot. Error bars represent SD. *P < 0.05; **P < 0.01; ***P < 0.001.

To explore the roles of ORP5 in RCC cells, we carried out several function experiments to investigate how functional properties of cells related to cancer progressions, such as migration and invasion, were altered when protein levels of ORP5 were changed. The results of wound healing and transwell assays showed that upregulation of ORP5 facilitated cell migration and invasion, while knockdown of ORP5 suppressed cell migration and invasion (Fig. 2A, B, C). Then, we investigated the effects of ORP5 on EMT markers. As shown in Fig. 2D, overexpression of ORP5 resulted in increased N-cadherin, Fibronectin, Vimentin, and decreased E-cadherin expression, and vice versa. The above results indicated that ORP5 promoted the migration and invasion of RCC cells in vitro.

### ORP5 regulates human RCC cells migration and invasion via regulating c-Met

**Fig. 3:**
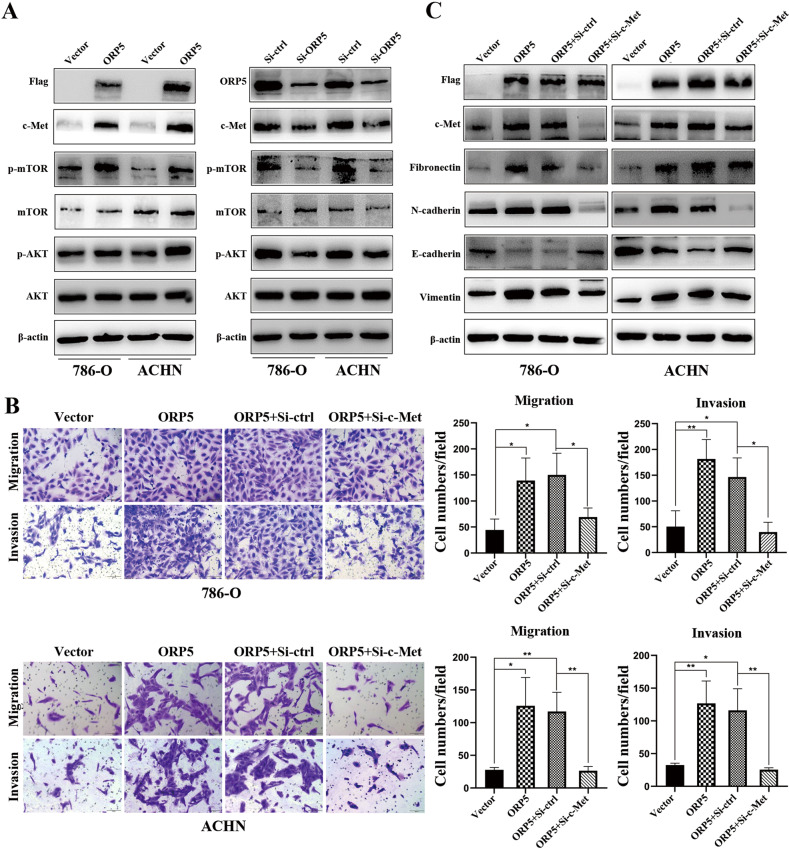
ORP5 regulates human RCC cells migration and invasion via regulating c-Met. **A** The expressions of Flag, c-Met, ORP5, p-mTORC1, mTORC1, p-AKT and AKT in RCC cells after ORP5 knockdown or ORP5 overexpression were tested by Western blot. **B** Representative images of transwell migration and invasion assays for ORP5-overexpressing ccRCC cells treated with siMet or siCtrl. Original magnifications, ×100 for (**B**). **C** The protein levels of EMT markers were examined by Western blot. Error bars represent SD. *P < 0.05; **P < 0.01; ***P < 0.001.

HGF/c-Met signaling pathway was one of the classical signaling pathways regulating the EMT process [14, 15]. Previous studies demonstrated that in HeLa cells ORP5 was involved in activating the mTORC1/AKT signaling pathway, a core signaling pathway of c-Met [16]. Additionally, our data indicated that the protein levels of phosphorylated mTOR (p-mTOR) and phosphorylated AKT (p-AKT) were increased after ORP5 overexpression in RCC cells and decreased in ORP5 knockdown cells, which indicated that ORP5 could activate the mTORC1/AKT signaling pathway in RCC cells (Fig. 3A). Therefore, we hypothesized that ORP5 could modulate EMT by regulating c-Met. As expected, our results showed that c-Met expression was positively associated with ORP5 (Fig. 3B). To further explore our hypothesis, rescue experiments using siRNA targeting c-Met were carried out to investigate whether ORP5 promoted migration, invasion, and EMT in RCC cells by upregulating c-Met expression. As is shown in Fig. 3C, knockdown of c-Met effectively restored the increase in aggression and migration of tumor cells caused by ORP5 overexpression. Besides, the results of western blot indicated that downmodulation of c-Met effectively restored the altered protein levels of EMT markers (Fig. 3D).

### ORP5 overexpression suppresses lysosomes degradation of c-Met

**Fig. 4:**
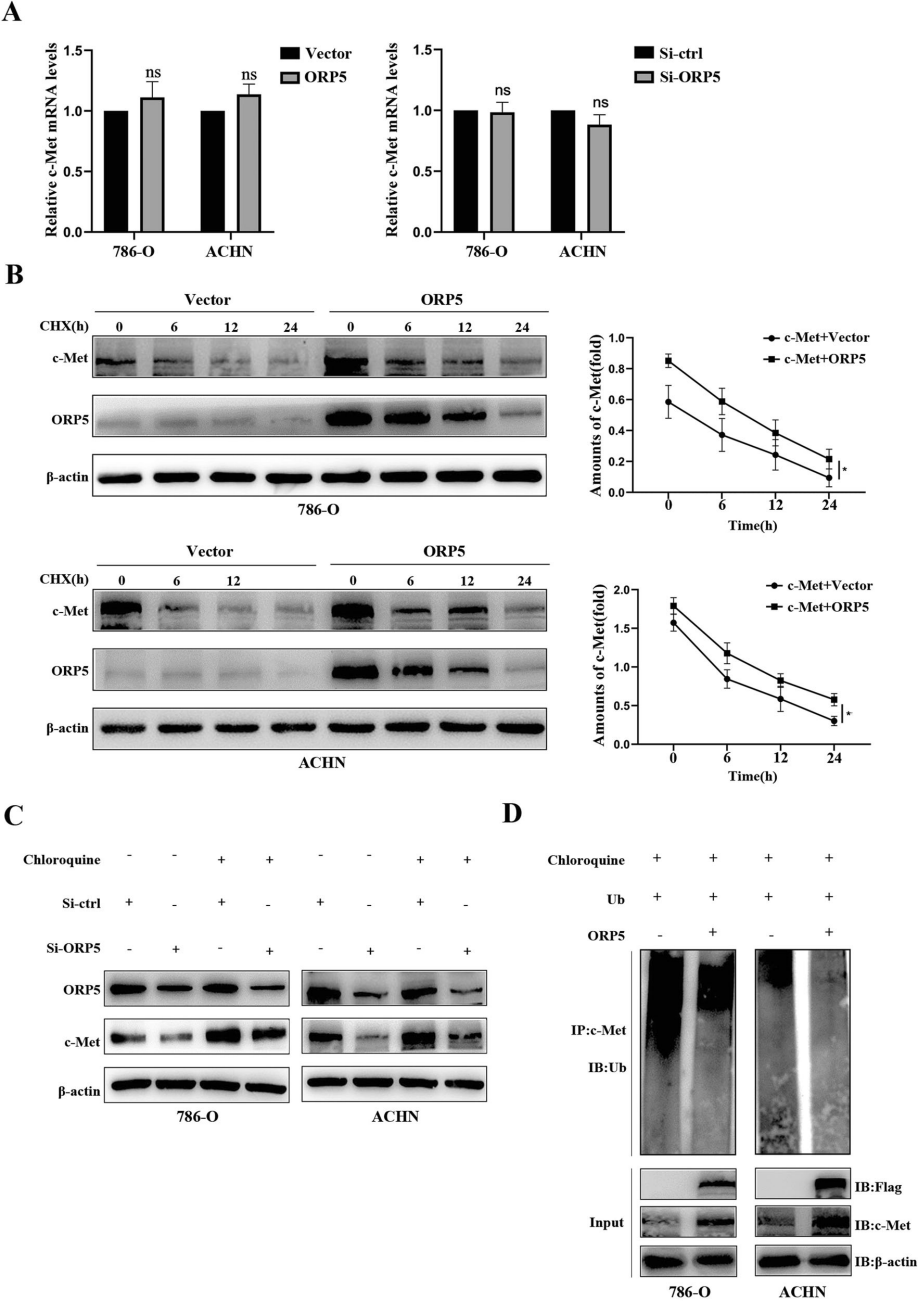
ORP5 overexpression suppresses lysosomes degradation of c-Met. **A** Q-PCR analysis was carried out to examine c-Met mRNA levels after ORP5 overexpression and knockdown in RCC cells. **B** Western blot was carried out to assess the effects of overexpressing ORP5 on the half-life of c-Met in 786-O, ACHN cells treated with cycloheximide (CHX) at 20 µg/ml and collected at 0, 6, 12, and 24 h. **C** Western blot of c-Met expressions in the indicated cells treated with or without Chloroquine (100 µmol). **D** Immunoprecipitation and immunoblotting were performed to analyze the ubiquitination of c-Met when co-transfected with Ub and ORP5 expression plasmids in these two RCC cells. Error bars represent SD. *P < 0.05; **P < 0.01.

Next, we sought to explore the possible mechanisms by which ORP5 regulated c-Met. Primarily, we found no significant changes in c-Met mRNA levels when ORP5 was knocked down or upregulated in RCC cells, indicating that ORP5 regulated c-Met not by affecting c-Met mRNA expression (Fig. 4A). We then postulated that the upregulation of c-Met in ORP5-overexpressing RCC cells might be due to the inhibition of c-Met degradation by ORP5. Consistent with our speculation, when we administered 786-O and ACHN cells with the protein synthesis inhibitor CHX at the corresponding interval, we found that the protein stability of c-Met in RCC cells expressing ORP5 was increased (Fig. 4B). Accordingly, we concluded that ORP5 could restrain the degradation of c-Met. To investigate how ORP5 affected the degradation of c-Met, Chloroquine was used to treat cells to depress lysosomal activity. The results indicated that Chloroquine treatment reversed the loss of c-Met (Fig. 4C). Furthermore, considering that c-Met could also be degraded by proteasomes, a proteasome inhibitor (MG132) was used to treat cells. The results showed that protein levels of c-Met could not be recovered (Fig. S1), thus indicating that ORP5 did not compromise c-Met degradation through proteasome activity. In addition, we performed a ubiquitin assay to verify whether ORP5 affected the ubiquitination of c-Met. The results indicated that ORP5 indeed suppressed the ubiquitination of c-Met (Fig. 4D).

### ORP5 decreases c-Cbl expression by promoting ubiquitination and proteasomal degradation

**Fig. 5:**
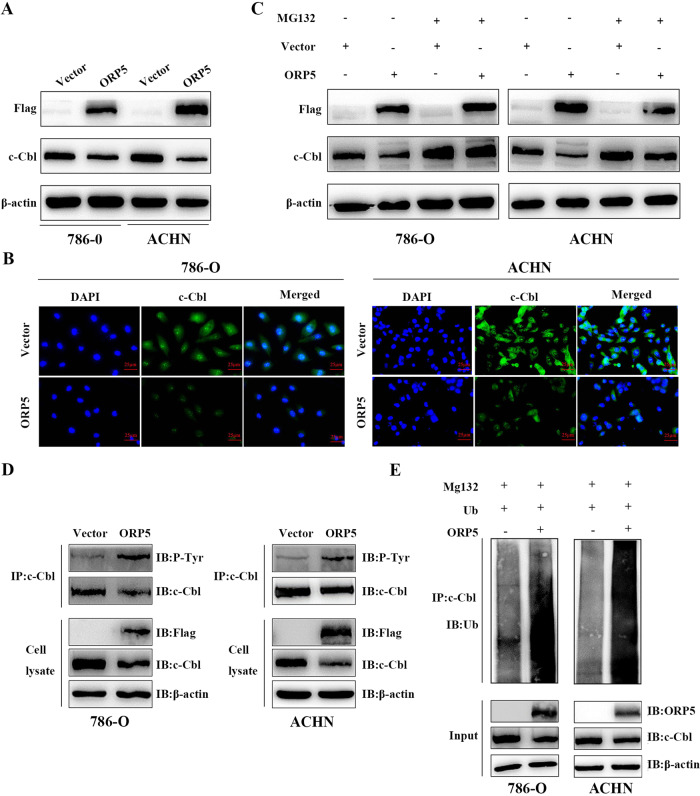
ORP5 decreases c-Cbl expression by promoting ubiquitination and proteasomal degradation. **A** Western blot of c-Cbl expressions after ORP5 upregulation in RCC cells. **B** IF staining of c-Cbl in ORP5-upregulated 786-O and ACHN cells and their corresponding control cells. Scar bar, 25 µm. **C** Western blot analysis of c-Cbl expressions in ORP5-upregulated RCC cells treated with or without MG132 (100 µmol). **D** The two RCC cells stably expressing ORP5 or empty vector were subjected to 24 h starvation in serum-free medium, immunoprecipitated (IP) with anti-c-Cbl antibody, and their phosphorylation status were confirmed by Western blotting with anti-phosphotyrosine antibodies (P-Tyr). **E** Immunoprecipitation and immunoblotting were performed to visualize the ubiquitinated c-Cbl proteins in ORP5-overexpressed 786-O and ACHN cells.

As ORP5 not directly interacted with c-Met (Figure S2), we postulated that ORP5 affected the ubiquitination of c-Met via other molecules. As an E3 ligase of c-Met, c-Cbl has a major role in the ubiquitination of c-Met. Activated c-Met interacts with c-Cbl to promote the ubiquitination of internalized c-Met and its degradation in the lysosome. The results of Immunofluorescence and Western blot both suggested that ORP5 suppressed the expression of c-Cbl (Figs. 5A, B). Then, the Western blot results showed that the addition of the MG-132 restored the ORP5-mediated decrease in c-Cbl expression (Fig. 5C). Meanwhile, we found that ORP5 could promote tyrosine phosphorylation of c-Cbl (Fig. 5D). Finally, the ubiquitin experiments were carried out to determine that ORP5 indeed promoted the ubiquitin process of c-Cbl (Fig. 5E).

### ORP5 promotes RCC metastasis in vivo through upregulating c-Met expression

**Fig. 6:**
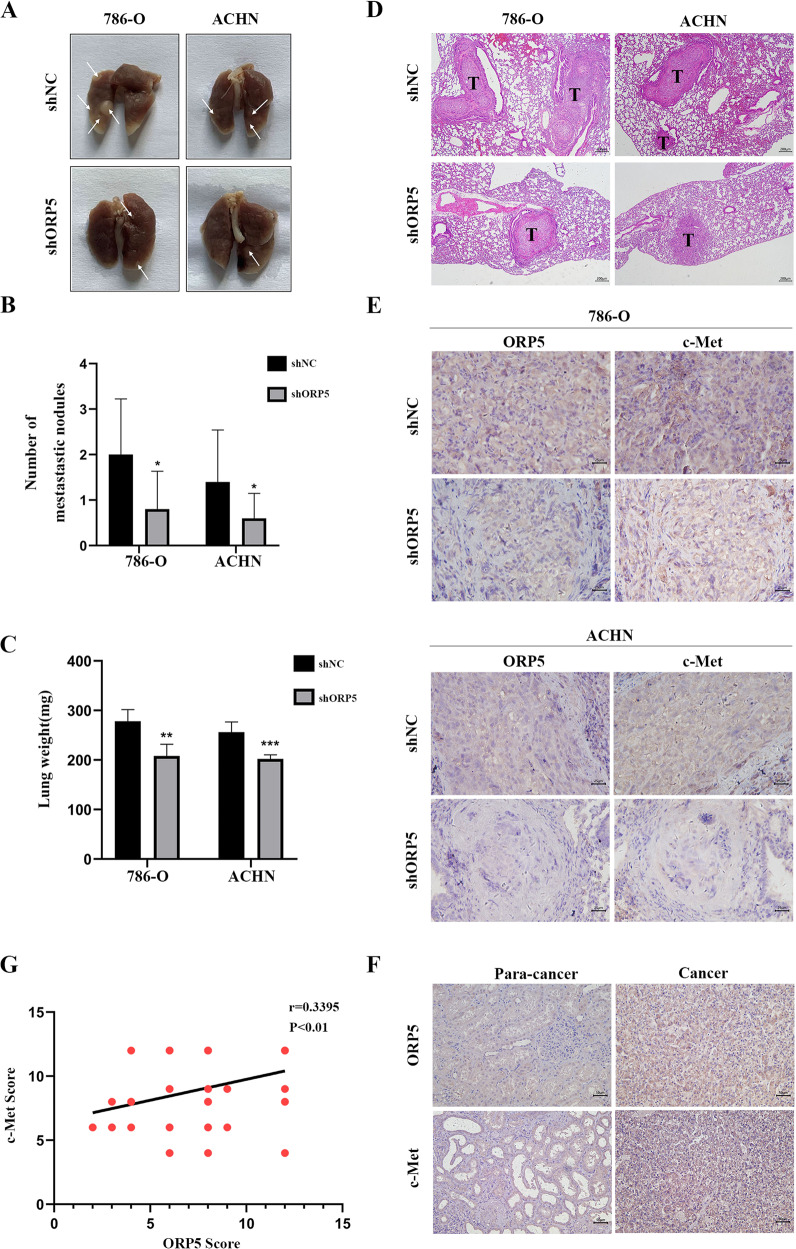
ORP5 facilitates metastasis of RCC in vivo through upregulating c-Met expression. **A** 2 × 10^6^ 786-O and ACHN cells transfected with LV-shORP5 and LV-shNC were injected into the tail vein of randomly grouped nude mice, respectively (*n* = 5). At 35 days after injection, the nude mice were sacrificed, then the lungs were removed and photographed. The white arrows pointed to metastatic nodules. **B** The number of metastatic nodules in the lungs of each mouse was counted. **C** Weights of the lungs were recorded. **D** H&E staining was performed on lung sections. Scale bar, 200 μm. **E** Representative pictures of IHC staining of ORP5, c-Met in mice lung tissues. Scale bar, 25 μm. **F** Representative IHC images of ORP5 and c-Met in ccRCC tissues. Scale bar, 50 μm. **G** The relationship between ORP5 IHC score or c-Met IHC score in ccRCC tissues was analyzed by Pearson correlation analysis. Error bars represent SD. *P < 0.01; ***P < 0.001.

To further determine whether ORP5 promoted tumor metastasis of RCC cells in vivo, we injected shNC, 786-O-shORP5 cells and ACHN-shORP5 cells into the nude mice via tail vein to establish a mouse model of cancer metastasis. The lungs were removed from the mice after thirty-five days and photographed. As is shown in Fig. 6A, B, the mice inoculated with shNC group had more metastatic nodules in the lungs than the shORP5 group. Besides, the lung weight of the shNC group was also greater than that of the shORP5 group (Fig. 6C). H&E staining indicated that the lung nidi of the mice were metastatic tumors (Fig. 6D). In addition, IHC staining of these metastatic nodules excised from nude mice indicated that nodules in the shNC group showed enhanced expression of ORP5 and c-Met compared to the shORP5 group. Meanwhile, the results verified a positive correlation between ORP5 and c-Met levels (Fig. 6E). More importantly, the expressions of c-Met and ORP5 were detected on ccRCC tissue chips with IHC assay. The data were further evidence that the expressions of c-Met and ORP5 in ccRCC tissues were positively related (Fig. 6F, G). In brief, upregulation of ORP5 promoted RCC metastasis by upregulating c-Met in vivo.

## Discussion

Abnormal expression of ORP5 and its role in cancer progression have been reported in several cancers [7, 8, 16, 26]. In this study, we demonstrated the roles of ORP5 in kidney cancer. First, ORP5 was overexpressed in RCC cell lines and tissues, and its high expression was closely related to tumor progression. Second, ORP5 overexpression significantly promoted RCC cells migration and invasion. According to the results, it was reasonable to assume that ORP5 might act as an oncogene in the progression of kidney cancer.

EMT is known to promote tumor progression by providing enhanced migration, invasive and survival properties to tumor cells. Our data showed that ORP5 facilitated EMT process by increasing N-cadherin, Vimentin and Fibronectin expressions and inhibiting E-cadherin protein expression, indicating that ORP5 could promote EMT and thereby promote tumor migration. Moreover, EMT is considered to be regulated via multiple signaling pathways influenced by the tumor microenvironment [27]. More specifically, receptor tyrosine kinase signaling plays a key role in regulating this process [28]. The c-Met can cooperate with some other kinds of tyrosine kinases to promote some classical signaling pathways such as PI3K/AKT and Ras/MAPK, thereby regulating tumor invasion, metastasis and EMT process [11, 29]. In 2018, Du et al. reported that ORP5 could activate mTORC1 signaling in HeLa cell. And, our results indicated that ORP5 could participate in activating mTOR/AKT signaling in RCC cells, which was one of the most important pathways of c-Met. Therefore, we hypothesized that ORP5 regulated the EMT process by regulating c-Met, and thus the migration and invasion of RCC cells. The results demonstrated that ORP5 indeed had an effect on the c-Met expression. Besides, in RCC tissue microarrays, ORP5 was positively related to c-Met (*r* = 0.3395, *P* < 0.01). Furthermore, we found that the use of siRNA targeting c-Met could reverse the increased migrative and invasive ability and the altered EMT markers of RCC cells caused by ORP5 upregulated. The above results indicated that ORP5 facilitated the malignant behaviors of RCC cells by upregulating c-Met.

Then, we further explored the potential mechanisms by which ORP5 regulated the protein levels of c-Met in kidney cancer cells. Our results indicated that down- or upregulation of ORP5 had no significant effect on the mRNA levels of c-Met. Then, we considered that ORP5 affected the degradation of c-Met and the results demonstrated that ORP5 did increase c-Met protein stability. The c-Cbl is known to promote ubiquitination of activated c-Met receptors and their degradation in lysosomes or sorting to subcellular signaling microdomains [30, 31]. However, Gui et al. proposed that the SOCS1-mediated downregulation differed from Cbl-dependent modulation of c-Met expression in that SOCS1 reduced c-Met protein levels by facilitating K48-dependent polyubiquitination and proteasomal degradation [32]. Therefore, proteasome inhibitor (MG132) and lysosomal inhibitor (Chloroquine) were both used in our study. The results showed that MG132 could not restore the decrease in c-Met protein levels due to knockdown of ORP5, but Chloroquine could. Based on the results, we concluded that ORP5 suppressed lysosomes degradation of c-Met. Previous publications identified that ubiquitination played an essential role in RTK downmodulation through targeting receptors to the lysosome [33]. Our data suggested that ORP5 inhibited the ubiquitination of c-Met. These results demonstrated that ORP5 repressed lysosomal degradation of c-Met by suppressing its ubiquitination.

The findings that Cbl proteins were ubiquitin-protein ligases of receptors and that ubiquitination regulated sorting of receptors to lysosomes had significantly improved our understanding of the mechanisms of RTKs downregulation, where c-Cbl was the E3 ubiquitin ligase of c-Met. For example, Taher et al. reported that c-Cbl functioned as a negative modulator of HGF/Met signaling in B cells [34]. In non-small-cell lung cancer, Tan et al. demonstrated that ubiquitination of c-Met was decreased in c-Cbl mutant cells compared to wildtype cells [35]. Considerable evidence also pointed that some RTK-derived tumor proteins avoided downregulation through inefficient Cbl recruitment, loss of Cbl binding sites, Cbl degradation, or by forming fusion proteins that escaped lysosomal degradation. Our results demonstrated that ORP5 could reduce c-Cbl protein levels. In Gastric Cancer, Lai et al. not only identified Met-dependent c-Cbl protein deletion in MET- expanded gastric cancer cell lines as an alternative mechanism responsible for dysregulated signaling, but Met-dependent c-Cbl loss might also facilitate cross-talk by indirectly enhancing EGF receptor signaling [36]. Therefore, it was necessary to further explore the possibility that c-Met might also promote the stability of other tyrosine kinases by virtue of its overexpression, thus forming a platform of activated RTKs that collectively contributed to the enhancement of oncogenic signaling.

Studies have indicated that in addition to the ubiquitination of its substrates, c-Cbl can also auto-ubiquitinate, resulting in its degradation. Furthermore, in these reports, auto-ubiquitination of c-Cbl often occurred after its tyrosine phosphorylation [23–25]. Therefore, a reasonable speculation emerged based on the above information: ORP5 promoted ubiquitination and proteasomal degradation of c-Cbl. Consistently, our data showed that ORP5 promoted tyrosine phosphorylation of c-Cbl and facilitated its ubiquitin-proteasome degradation. However, it is unclear through which kinase ORP5 promotes the tyrosine phosphorylation of c-Cbl. It remains to be explored whether the high levels of c-Met due to c-Cbl degradation in turn promote tyrosine phosphorylation of c-Cbl and thus its ubiquitination, or whether ORP5 promotes tyrosine phosphorylation of c-Cbl via an unknown kinase?

**Fig. 7:**
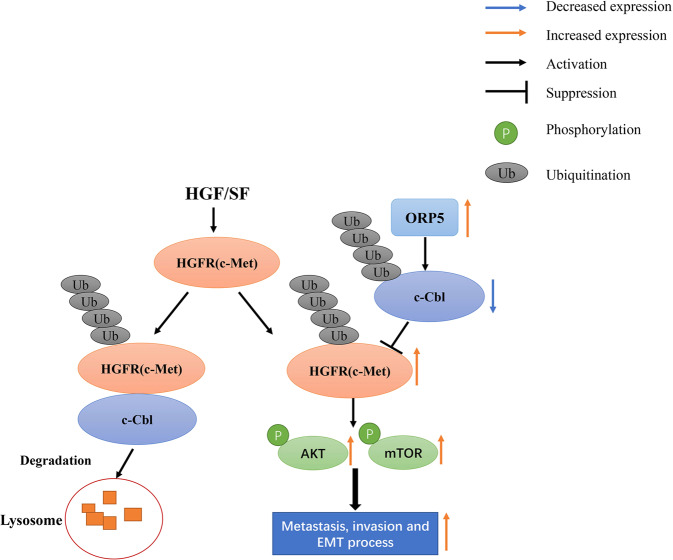
The graphic illustration of ORP5 modulation of c-Met expression and metastasis and invasion of RCC. ORP5 stabilizes the c-Met protein levels by promoting the ubiquitination of c-Cbl and subsequently inhibiting c-Met ubiquitination and lysosomal degradation.

In summary, we demonstrated that high expression of ORP5 was related to the development and progression of kidney cancer. Moreover, this study presented the mechanism by which ORP5 promoted the progression of RCC: ORP5 promoted c-Cbl ubiquitination and decreased its protein level, thereby inhibiting c-Met ubiquitination and lysosomal degradation. The abnormal increase of c-Met promoted the EMT process and ultimately facilitated the metastasis of renal cell carcinoma (Fig.7).

## Materials and methods

### Antibodies and reagents

Antibodies specific for c-Met (#25869-1-AP); c-Cbl (#25818-1-AP); Fibronectin (#1G10F9); Vimentin (#10366-1-AP); E-cadherin (#20874-1-AP); Tubulin (#10094-1-AP); DYKDDDDK (#66008-3-Ig); β-actin (#66009-1-Ig); mTOR (#20657-1-AP) were obtained from proteintech (Wuhan, China). The Phospho-AKT (Ser473) (#4060) and AKT (#9272) antibodies were both supplied by Cell Signaling Technology (Danvers, MA). An anti-phosphotyrosine antibody was obtained from Abbkine. Antibodies used were goat polyclonal to ORP5 (Abcam, ab59016); mouse monoclonal to N-cadherin (Servicebio, GB12135); p-mTOR (59. Ser 2448) (Santa Cruz Biotechnology, sc-293133). For immunoblotting, horseradish peroxidase-conjugated secondary antibodies were obtained from Beyotime. For immunofluorescence, CoraLite488––conjugated Affinipure Goat Anti-Mouse IgG(H + L) was obtained from proteintech.

Chloroquine Sulfate and MG-132 were from APExBIO (MA, USA). Cycloheximide (CHX) was from MedChemExpress (shanghai, China).

### Cell culture

The human RCC cell lines Caki-1, ACHN, 786-O, and normal human renal tubular epithelial cell HK-2 were all provided by the Cell Bank of the Chinese Academy of Sciences (Shanghai, China). The cell lines were recently tested for mycoplasmacontamination and authenticated by STR profiling, then maintained in DMEM medium (Gibco, Grand Island, NY, USA) with the addition of 1% antibiotics (Beyotime) and 10% FBS (Gibco). The cells mentioned above were maintained at 37 °C with 5% CO_2_ in a humidified cell incubator.

### SiRNA, plasmids transfection and stable cell lines construction

Small interference RNAs (siRNAs) targeting human ORP5 (siORP5), human c-Met siMet) and negative controls (siCtrl) were obtained from GenePharma (Shanghai, China). The siRNA sense sequences were 5ʹ-GCGGAGACAAUGAGCUCUATT-3ʹ for siORP5, 5ʹ-AUUUUCUGUAAUCAAAUGATT-3ʹ for siMet and 5ʹ-UUCUCCGAACGUGU-CACGUTT-3ʹ for siCtrl. For siRNAs transfections, siLentFect™ Lipid Reagent (Bio-Rad, Hercules, CA, USA) was used. For transient transfections, the human ORP5 plasmid was cloned into the pcDNA3.1-3xFlag-C vector (YouBia) and Hieff Trans™ Liposomal Transfection Reagent (YEASEN, shanghai, China) was used to transfect empty vectors and pcDNA3.1-ORP5 plasmids into the RCC cells. Cells were used for the following experiments after transfection for 24 h or 48 h. To obtain stable cell lines, the two RCC cells were infected with lentivirus-shORP5 and lentivirus-shNC (GenePharma) and selected using 1 μg/ml puromycin (Vicmed, Xuzhou, China) for 14 days.

### Tissue microarrays and immunohistochemistry assays

The tissue microarrays (TMAs) were provided by Shanghai Outdo Biotech Company, containing 75 RCC patient tissues. The study was supported by the Ethics Committee of Shanghai Otto Biotechnology Co (HKidE150CS03).

Firstly, the tissue microarrays were deparaffinized at 65 °C for 1.5 h, then re-dewaxed with dimethylbenzene. Next, the sections were heated in boiling 0.01 M sodium citrate buffer for two minutes to conduct antigen retrieval. To avoid nonspecific staining, 10% normal goat serum was used to block the tissue microarrays for 1 h. Then, the tissue chips were incubated with corresponding primary antibodies at room temperature for 2 h. After flushing with phosphate-buffered saline (PBS), the tissue microarrays were incubated separately with secondary antibodies and then the staining was performed with the DAB detection kit (Zhongshan biotech, Beijing, China), according to the manufacturer’s protocols. Lastly, the tissue microarrays were stained with hematoxylin and sealed with neutral resins. During the experiments, four samples fell off the chip. Therefore, there were only 71 samples in subsequent statistical analysis.

ORP5 staining images were assigned as the IHC score, which was computed by multiplying the intensity score and the range score together, where the intensity score was (0 = negative; 1 = weak; 2 = moderate; 3 = strong) and the score of the extent of positive stained cells was (1 = 0–25%; 2 = 26–50%; 3 = 51–75%; 4 = 76–100%). The staining pattern was defined as low (0–6) and high (8–12) according to the scores.

### Transwell Assays

The transwell chambers with 8.0 μm pores (BD Bioscience, San Jose, CA, USA) were used for invasion and migration assays. In general, the cells dispersed in 200 μl serum-free culture medium were spread into the upper chamber and 600 μl medium with 10% FBS was inserted in the lower chamber. What calls for attention was that the Matrigel (Corning Incorporated) needed to be put in the chambers in advance when the cell invasion assay was done. After 24 h of culture for migration and 48 h for invasion, the cells that did not pass on the upper insertion membrane were wiped with PBS and the cells that passed on the lower insertion membrane were stained using 1% crystalline violet dye solution, examined and photographed under light microscopy. Each experiment was executed in triplicate.

### Wound healing assay

The two RCC cells were seeded and grown overnight to reach about 95% confluence in a 6-well plate. The next day, we used a sterile 1 ml pipette tip to scratch the cell monolayer with the advisable force. Then, the cells were cultured again after removing cellular debris with the fresh medium. Images of the wound were collected at 0 and 48 h. The experiment was performed independently in triplicate.

### Western blot assay

The total protein of RCC cells was extracted with RIPA lysis buffer with fresh protease and phosphatase inhibitor cocktail. A bicinchoninic acid (BCA) Kit (Beyotime Biotechnology) was used to detect protein concentration. Next, 20 μg protein samples were electrophoresed by 7.5% SDS–PAGE and then transferred to nitrocellulose membranes (Pall Corporation). After blockade in 5% skim milk for 1 h at room temperature, the membranes were incubated at 4 °C overnight with the corresponding antibodies. The next day, the membranes were rinsed with washing buffer and then incubated with secondary antibodies. After rewashing the membranes, the signal was detected by a sensitive chemiluminescence detection system.

### Co-IP assays and ubiquitination assays

The method of extracting the whole-cell lysate used for Co-IP assays and ubiquitination assays was consistent with the method mentioned in Western blot assays. The whole cell lysate was then incubated with the detection antibody overnight at 4 °C. The next day, 50 µl of protein A/G beads (Beyotime) were added into the protein samples and remixed by rotation for 6 h. Lastly, the samples were detected by 7.5% SDS–PAGE electrophoresis after centrifugation to remove the supernatant.

### Immunofluorescence

The ACHN and 786-O cells were seeded on coverslips in 6-well plates (10,000 cells per well) and fixed using 4% paraformaldehyde. Cells were permeabilized with 0.5% TritonX-100 in PBS and blocked with 1% bovine serum albumin (BSA). Then, the cells were incubated with a primary antibody at 4 ˚C overnight. The second day, the cells were treated with Goat Anti-Mouse IgG(H + L) and CoraLite488 conjugate at room temperature for 1 h. Finally, the cells were observed via laser-scanning confocal microscopy (Nikon) after staining with 4′,6-diamidino-2-phenylindole (DAPI) (Vector Laboratories).

### Tail vein cancer metastasis model

Twenty female BALB/cA-node mice (aged 4–5 weeks) were approved by Beijing Huafukang Bioscience (Beijing, China) (randomly divided into different groups with four mice in each group). RCC cells with ORP5 stably knockdown or the negative control cells (2 × 10^6^ cells in 0.1 ml of PBS) were injected into the 6 weeks-old female nude mice via their tail vein (*n* = 5/group). The lungs with metastatic foci were excised, and photographed after five weeks. The number of metastatic tumors in each lung was counted and the lung weights were recorded. Lastly, hematoxylin-eosin staining (H&E) and IHC assay were performed on the lung tissues.

### Statistical analyses

All statistical analyses and data plotting were executed with GraphPad Prism 8. The Student’s *t*-test or two-way ANOVA were used to analyze the differences between groups. In RCC tissues, relationships between ORP5 expression and clinicopathological features were analyzed by Pearson’s Chi-square test or Fisher’s exact test, while the correlation between ORP5 and c-Met IHC score was analyzed by Pearson’s correlation analysis. All data were shown as the mean ± standard deviations (SD). *P* values < 0.05 were considered statistically significant.

## Supplementary information

S3

S1

S2

## Data Availability

The datasets used and analyzed during the current study are available from the first author or corresponding author upon reasonable request.
