# Supplementary figures and images for: ORP5 promotes tumor metastasis via stabilizing c-Met in renal cell carcinoma
Source: Cell Death Discov. 2022 Apr 21;8:219. doi: 10.1038/s41420-022-01023-3 (PMC9023482; doi:10.1038/s41420-022-01023-3)

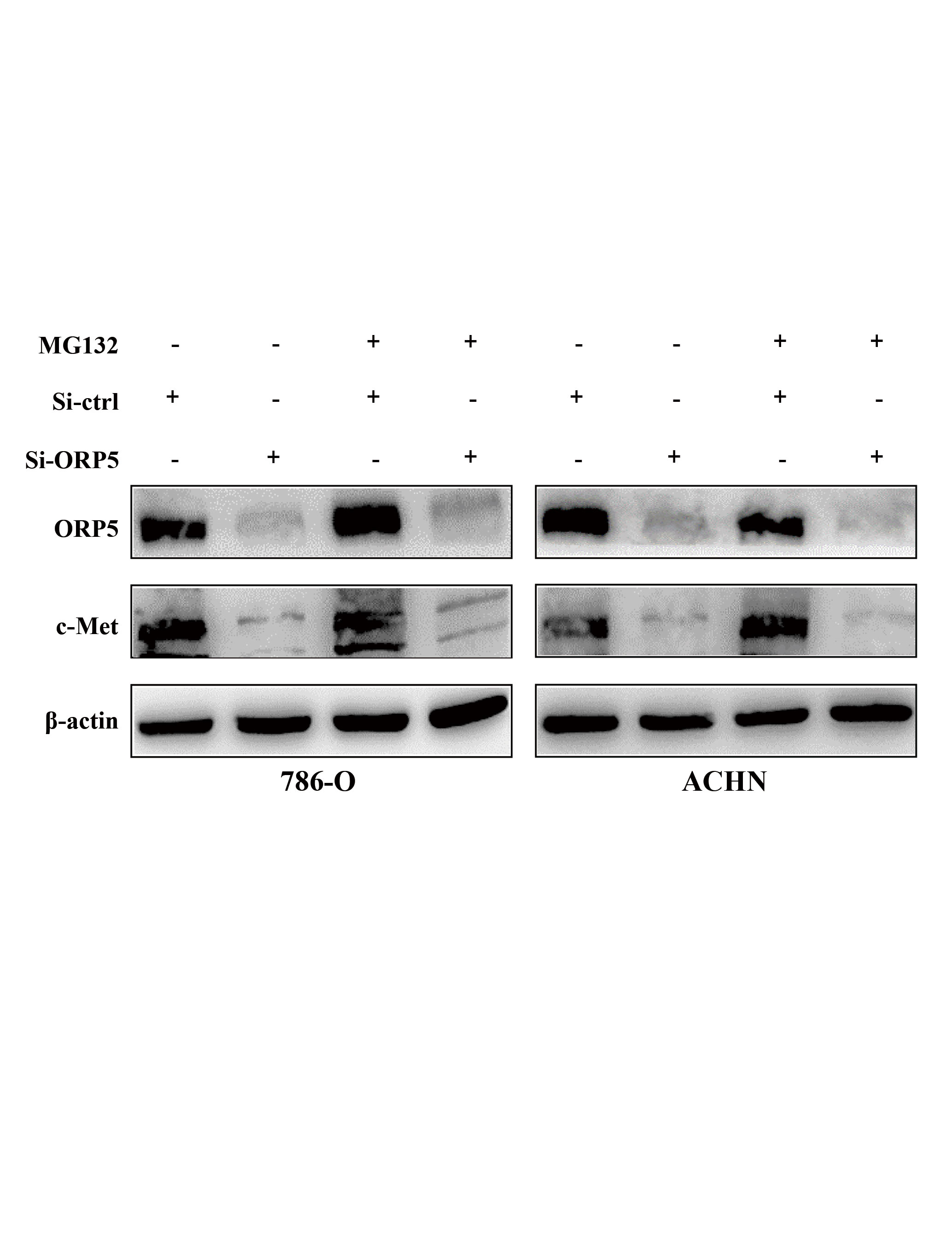

Supplement: Supplementary file 1 — S3 [file 41420_2022_1023_MOESM1_ESM.jpg]

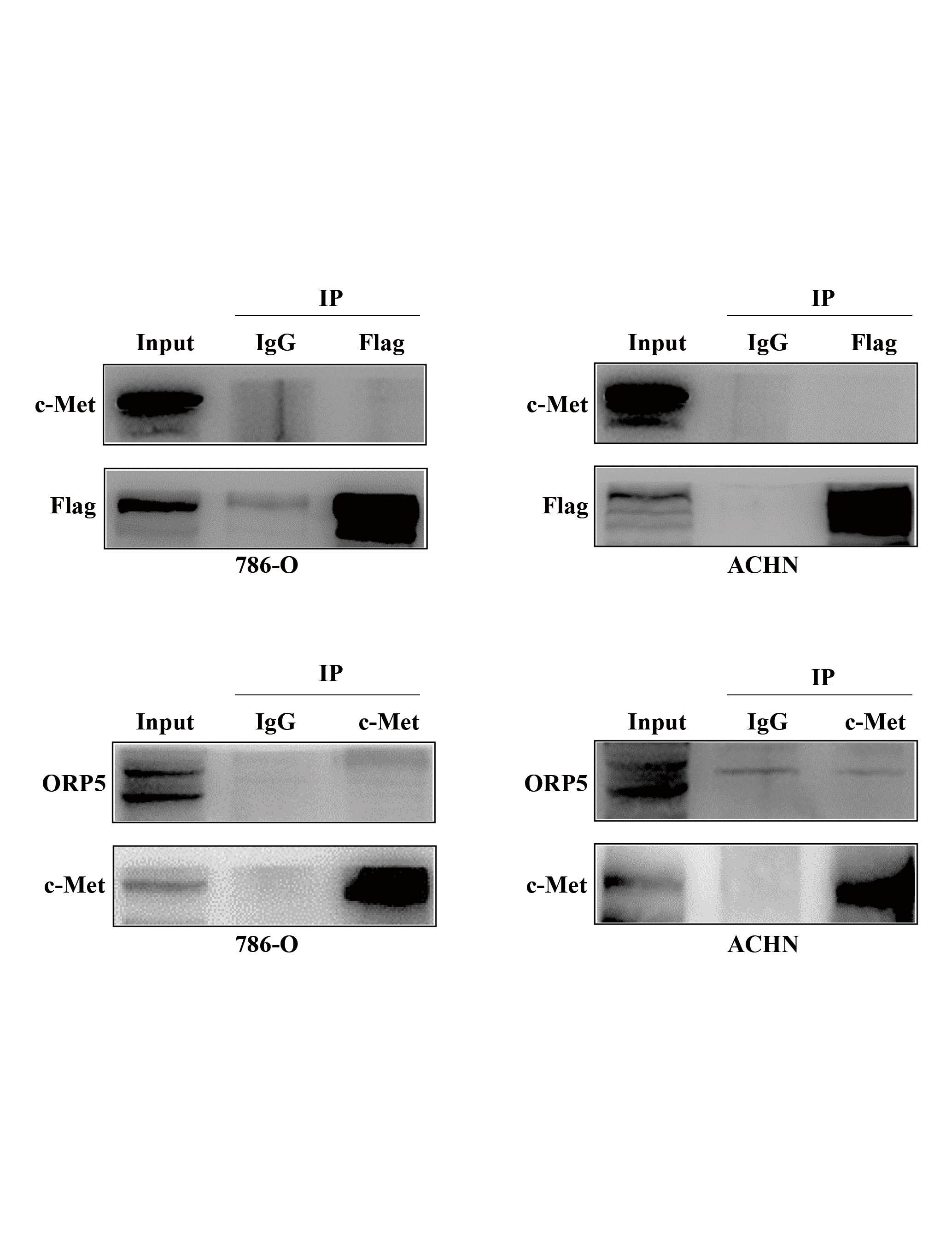

Supplement: Supplementary file 2 — S1 [file 41420_2022_1023_MOESM2_ESM.jpg]

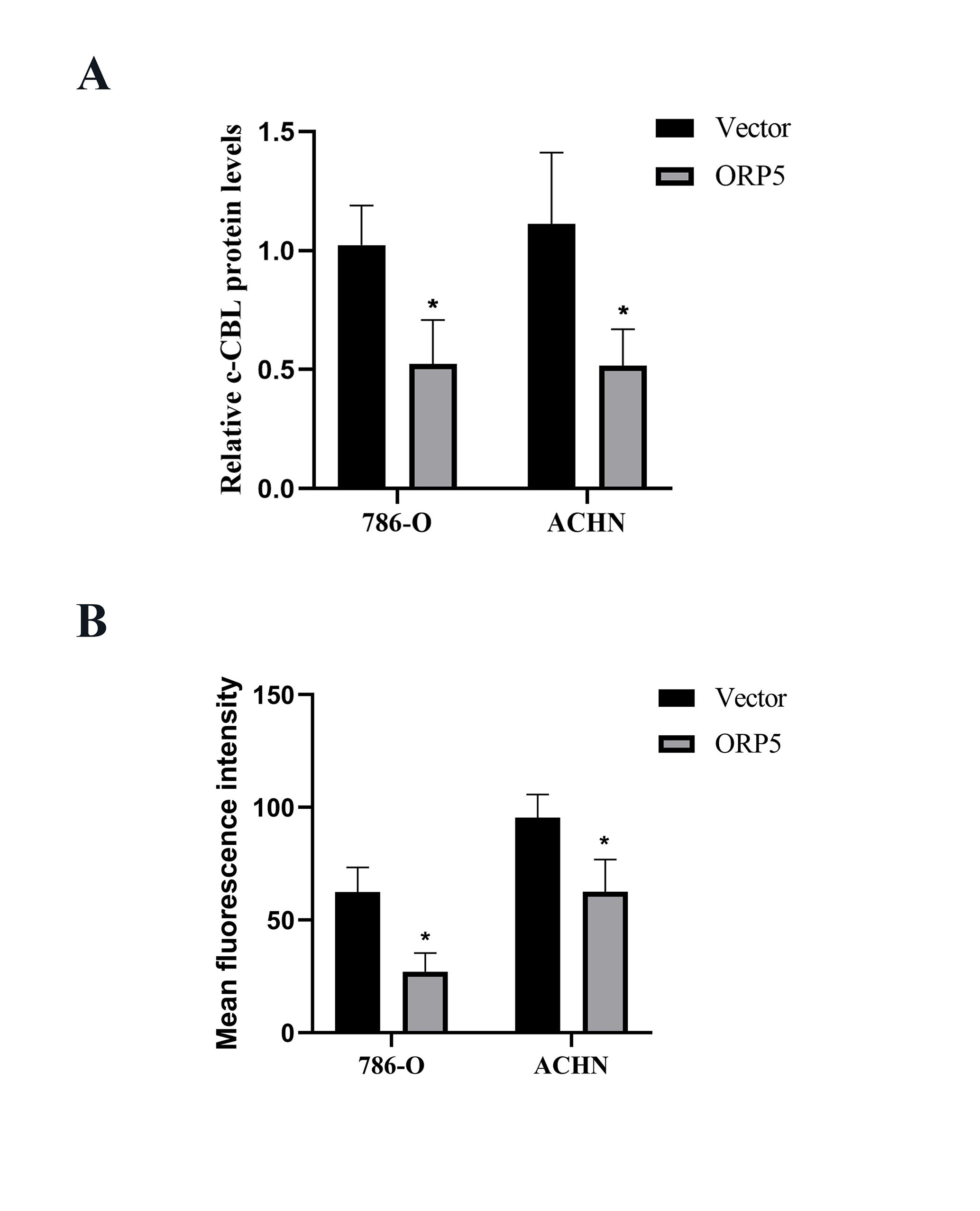

Supplement: Supplementary file 3 — S2 [file 41420_2022_1023_MOESM3_ESM.jpg]
